# Live-Cell Imaging of Flaviviridae Family Virus Infections: Progress and Challenges

**DOI:** 10.3390/v17060847

**Published:** 2025-06-13

**Authors:** Siena M. Centofanti, Nicholas S. Eyre

**Affiliations:** College of Medicine and Public Health, Flinders University, Bedford Park, SA 5042, Australia; cent0038@flinders.edu.au

**Keywords:** live-cell imaging, fluorescence microscopy, orthoflavivirus, HCV, virus replication

## Abstract

The ability of a virus to be propagated within a host cell is dependent on a multitude of dynamic virus–host interactions. Live-cell imaging is an invaluable approach in the study of virus replication cycles and virus–host interactions as it can allow for the direct visualisation of key events and interactions in real time. These details can provide unique insights into many aspects of viral infections including the cellular pathways that are exploited by viruses, the evasion of host immune defences, and viral pathogenesis. This review summarises the live-cell fluorescence imaging approaches that have been developed and applied to study *Flaviviridae* virus family members that are responsible for significant public health burdens and outbreaks which, in many instances, are increasing in frequency and severity. We discuss how these approaches have expanded our understanding of fundamental stages of viral replication cycles by enabling the direct visualisation of the localisation, trafficking, and interactions of virus particles, proteins, and genomes at distinct stages. The strategies that can be employed to enhance the biological relevance of live-cell fluorescence imaging acquisitions are discussed, along with how live-cell imaging approaches can be further developed to increase resolution, enable multi-colour imaging, and support the long-term visualisation of multiple stages of a viral replication cycle.

## 1. Introduction

The *Flaviviridae* virus family of enveloped positive-sense single-stranded RNA (+ssRNA) viruses is composed of the following four genera: Orthoflavivirus, Hepacivirus, Pegivirus and Pestivirus. Many members of the Orthoflavivirus and Hepacivirus genera are responsible for causing significant disease in humans. For example, hepatitis C virus (HCV), the founding member of the *Hepacivirus* genus, remains a leading cause of chronic hepatitis, liver cirrhosis, and hepatocellular carcinoma worldwide due to the absence of a prophylactic vaccine and despite the availability of effective antivirals [1]. Medically significant orthoflaviviruses include dengue virus (DENV), Japanese encephalitis virus (JEV), West Nile virus (WNV), Zika virus (ZIKV), yellow fever virus (YFV), and tickborne encephalitis virus (TBEV). These viruses are spread by mosquitos or ticks throughout tropical and subtropical regions of the world and cause a broad range of symptoms including haemorrhagic fever, encephalitis, meningitis, and neurological impairment in newborns. The incidence of many orthoflavivirus infections and outbreaks has risen sharply in recent decades due to climate change-driven vector spread, population growth, unplanned urbanisation in endemic regions, and increased travel to these areas [2,3]. Currently, there are no antiviral therapies available for orthoflaviviruses, and safe and effective vaccines are only broadly available for JEV, YFV, and TBEV. For many orthoflaviviruses vaccine development is hindered by challenges associated with the potential for antibody-dependent enhancement (ADE) of viral infection, driven by co-circulation and serological cross-reactivity among closely related viruses [4].

As obligate intracellular pathogens with small genomes and thus a limited coding capacity, viruses interact with a broad range of host proteins to hijack the cellular machinery required to carry out each stage of the viral replication cycle and evade host innate immune responses and intrinsic restriction factors [5]. Each stage of the viral replication cycle consists of complex, dynamic, and tightly regulated sequences of molecular events. Efforts to understand these events and how they are governed by virus–host interactions have enhanced our understanding of viral replication, pathogenesis, manipulation of host cell machinery, and antiviral immunity. The most widely used imaging approach to study viral infections is fixed-cell fluorescence imaging, where cells are commonly treated with fixatives to preserve their structure at a specific time before direct or indirect staining with fluorophore-conjugated antibodies or probes that are directed towards relevant viral or host cell factors [6]. This approach is well suited to the analysis of co-localisation between viral and host cell factors, determination of the cellular localisation of viral factors, and the analysis of virus-induced changes in the morphology of cellular compartments. Alternatively, virus-infected cells can be fixed and processed for electron microscopy, which enables the visualisation of virus particles and cellular compartments with nanometre resolution to reveal ultrastructural details [6]. Unlike fixed-cell imaging, live-cell imaging provides real-time, spatiotemporal insights into viral replication cycles by visualising the localisation and trafficking of viral particles, proteins, and genomes in relation to host cell factors, organelles, and structures. However, there is often a trade-off between resolution and phototoxicity, which can impact the quality of live-cell imaging data and the biological insights that can be revealed. This review summarises the live-cell imaging approaches and tools that have been developed and applied to the study of infections with members of the Orthoflavivirus and Hepacivirus genera (Table 1) and discusses the respective strengths and limitations of these approaches (Table 2). These approaches include cellular reporter systems to detect viral infections, tracking single virus particles during entry with lipophilic dyes, and the visualisation of viral protein or RNA genome localisation, traffic, and interactions using genetically modified reporter viruses. Furthermore, we discuss the strategies that can be implemented to optimise live-cell imaging resolution, duration, and biological relevance, as well as emerging trajectories in these fields.

## 2. The Orthoflavivirus and Hepacivirus Genome Organisation and Life Cycle

**Figure 1:**
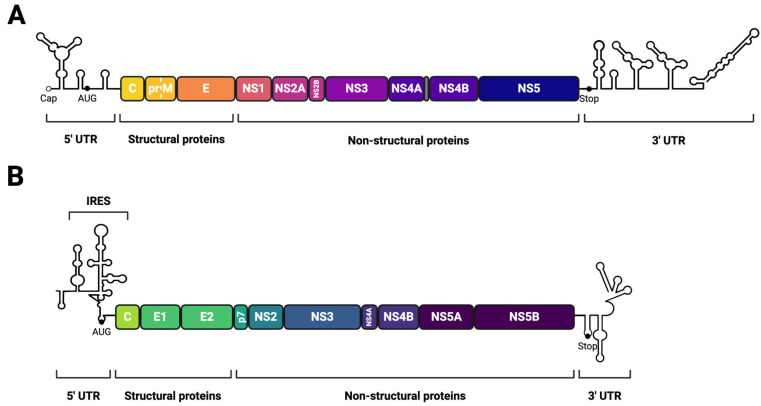
A schematic representation of the *Flavivirus* (**A**) and *Hepacivirus* (**B**) positive-sense, single-stranded RNA genomes. Both genomes are flanked by 5′ and 3′ untranslated regions (UTRs). The single open reading frame is translated into a polyprotein which is co- and post-translationally cleaved into three structural proteins and seven non-structural proteins. *Flavivirus* genomes feature a type-I cap within the 5′UTR, whilst *Hepacivirus* genomes feature a type-III internal ribosome entry site (IRES) within the 5′UTR.

**Figure 2:**
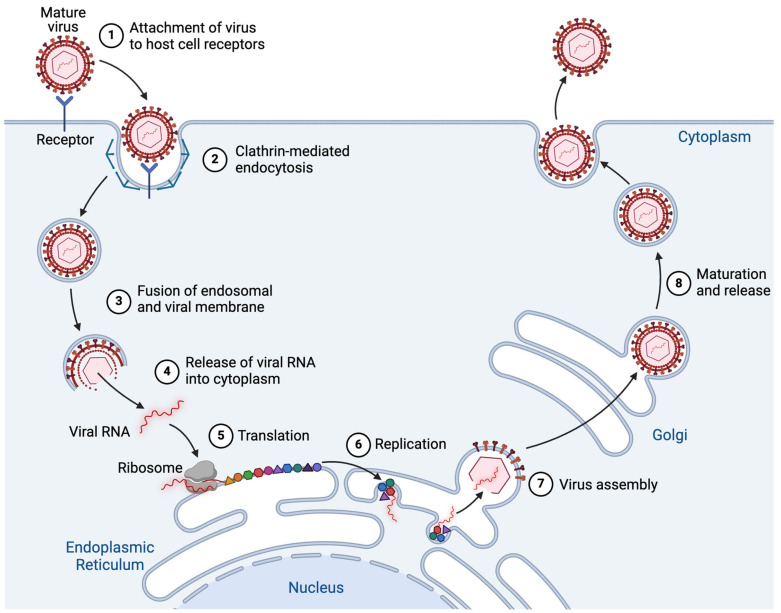
A schematic representation of the stages of the *Flaviviridae* life cycle.

Orthoflaviviral and hepaciviral genomes are positive-sense, single-stranded, non-segmented RNA molecules that are approximately 11 kB and 10 kB in length, respectively (Figure 1). These genomes consist of a single open reading frame (ORF) that is flanked by 5′ and 3′ untranslated regions (UTRs). The ORF encodes a polyprotein cleaved by viral and host cell proteases into three structural proteins, which mediate viral entry and particle formation, and seven non-structural proteins, which drive genome replication, membrane rearrangement, pathogenesis, and immune evasion. For members of the Orthoflavivirus and Hepacivirus genera, attachment of virus particles to susceptible host cells is mediated by the interaction of viral envelope glycoproteins with host cell surface receptors and allows virus particles to enter host cells via receptor-mediated endocytosis (Figure 2) [32,33]. Virus particles typically bind to several receptors with low affinity prior to engaging specific entry receptors that directly activate endocytosis. Late endosome acidification triggers the fusion of the endosomal and viral membrane which releases the viral genome into the cytoplasm [34,35]. The viral genome is translated by the host ribosome into a single ER membrane-associated polyprotein. This polyprotein is cleaved both co- and post-translationally by viral and host proteases to liberate the structural and non-structural viral proteins. Several non-structural proteins interact with host factors to drive the formation of ER membrane-derived replication organelles which serve as sites of viral genome replication [5]. The three-dimensional architecture and composition of these virus-induced membrane rearrangements have been resolved by several groups using electron tomography and immunofluorescence microscopy [30,36,37]. These studies suggest that the organelles concentrate viral proteins and host cell factors that are required for viral genome replication, protecting viral RNA from innate immune sensing, and spatially coordinating viral replication and assembly. Replication is facilitated by the viral replication complex (RC) formed by the association of the non-structural proteins with replication organelle membranes [5]. Progeny genomes either serve as templates for further translation or replication or exit the replication organelle to interact with capsid proteins, forming nucleocapsids. Nucleocapsids then acquire a lipid membrane and envelope through budding from microdomains within the ER membrane that are enriched with the viral envelope proteins [5]. The newly formed orthoflaviviral and hepaciviral particles undergo maturation within the secretory pathway. The maturation of orthoflaviviral particles is facilitated by the host protease furin which cleaves the pr peptide from the viral M protein within the Golgi, whereas HCV particle maturation is thought to depend upon apolipoprotein E (ApoE) [38,39]. Mature virus particles are then thought to be released from cells via the secretory pathway.

## 3. Monitoring Viral Replication and Spread Using Viral Protease-Dependent Reporters

Vector-based viral protease-dependent reporter systems that link viral proteolytic activity to a fluorescent signal enable single cell monitoring of infections via live-cell fluorescence microscopy. While reporter viruses encoding fluorescent proteins also allow for the live-cell imaging of infections, the insertion of a reporter tag within a viral genome often results in reduced fitness and genetic instability, making viral protease-dependent reporter systems a suitable alternative to study native strains and clinical isolates. Several groups have generated reporter systems that involve viral-protease dependent translocation of a fluorescent protein from the ER or mitochondria to the nucleus of infected cells [7,8,9,10]. A number of orthoflavivirus studies have utilised the proteolytic cleavage site between non-structural protein 4B (NS4B) and non-structural protein 5 (NS5) by expressing NS4B fused to the first 10 amino acids of NS5, a nuclear localisation signal (NLS) and a fluorescent protein or reporter enzyme (e.g., Cre recombinase) that can drive expression of a fluorescent protein [8,9,12,40]. In these systems, viral NS2B-3 protease-dependent cleavage of the NS4B-NS5 junction results in re-localisation of the fluorescent reporter or Cre recombinase to the nucleus to allow identification of infected cells via changes in nuclear fluorescence, either directly or via Cre-mediated reporter expression events. These systems have been applied to DENV and ZIKV infections and have enabled the visualisation of infection with clinically relevant viral isolates [8,12], the study of virus-induced apoptosis [9], and the characterisation of DENV-induced alteration of mitochondrial morphodynamics; specifically, the increased elongation and decreased motility of mitochondria as determined via live-cell imaging analyses [40]. While powerful, a potential complication of these systems is the impact of NS4B overexpression on host cell membrane rearrangements and host factors. This concern is supported by several studies showing that NS4B enhances pathogenicity through a variety of mechanisms, including the perturbation of mitochondrial morphodynamics, to inhibit innate immune signalling, induction of pro-inflammatory cytokine secretion from infected cells, and the induction of autophagy [41,42,43].

Recent studies have led to the development of versatile viral protease-dependent reporters that do not rely upon the expression of viral proteins for cytosolic retainment and proteolytic processing [7,11]. Arias-Arias et al. generated cytosolic *Flavivirus*-activatable green fluorescent protein (FlaviA-GFP) and mNeptune (FlaviA-mNeptune) reporters by fusing a hydrophobic quenching peptide to the C-terminus of each fluorescent protein via a linker containing a *Flavivirus*-conserved NS3 cleavage site [7]. Upon infection of cells stably expressing the reporters, the *Flavivirus* NS2B-NS3 protease cleaves the linker and the quenching peptide is released, allowing the fluorescent protein to undergo chromophore maturation and emit a fluorescent signal when appropriately excited. The FlaviA-GFP reporter enabled the sensitive detection of infections with DENV2 and ZIKV clinical isolates by live-cell imaging, whilst the FlaviA-mNeptune reporter exhibited a favourable signal-to-noise ratio when used in a fluorescent real-time plaque assay for these viruses. The use of FlaviA-mNeptune in combination with fluorescent dyes enabled the simultaneous detection of ZIKV infection, chromatin condensation, and cell death with single cell and single plaque resolution. More recently, Pahmeier et al. generated a DENV-specific reporter construct that employs the transmembrane (TM) domain of the ER resident protein sec16β as an ER anchor [7]. The sec16β transmembrane (TM) domain was connected to a GFP and nuclear localisation signal (NLS) via a variable linker region encoding a DENV NS2B-NS3 protease-specific cleavage site, which is cleaved by the proteases of all four DENV serotypes with high specificity. Accordingly, DENV infection or heterologous expression of the NS2B-NS3 protease results in cleavage-mediated relocation of GFP to the nucleus. Furthermore, as it is flanked by restriction enzyme recognition sites, the variable linker region of the reporter can be readily modified to encode protease cleavage sites recognised by the viral proteases of other positive-sense single-stranded RNA viruses.

Viral protease-dependent reporters that have been generated to detect HCV infections exploit the ability of the HCV NS3-4A protease to cleave the human mitochondrial antiviral signalling protein (MAVS) [10,13]. In an early study, reporters relying on the NS3-4A-mediated cleavage of MAVS to trigger fluorescent protein relocalisation were developed to detect infections by various HCV strains and were applied to the study of HCV entry receptors using multi-colour live-cell imaging [10]. Similarly, Ren et al. developed the NIrD (NS3-4A Inducible rtTA-mediated Dual-reporter) system where Huh-7.5(NIrD) stable cells express a fusion protein consisting of the reverse tetracycline-controlled transactivator (rtTA) and the C-terminal segment of MAVS encoding the HCV NS3/4A cleavage site (rtTA-MAVS [C]) [13]. Upon infection, the NS3/4A protease cleaves rtTA-MAVS(C), causing rtTA to translocate from the mitochondria to the nucleus. In the presence of doxycycline, rtTA activates a tetracycline responsive promoter to drive mCherry expression for simple identification of infected cells via fluorescence microscopy or flow cytometry.

## 4. Genetic Tagging of Viral Genomes for Analysis of Viral Protein Traffic and Virus–Host Interactions

Productive infections can be initiated by transfecting or electroporating cells with viral RNA generated by in vitro transcription from a cDNA clone encoding a reverse-transcribed full-length viral RNA genome (reviewed in [44]). The insertion of reporter genes within these cDNA clones by molecular cloning has enabled the generation of stable reporter viruses. These tools have been widely used to detect viral replication in vitro and in vivo, perform high-resolution imaging and quantitative analyses of viral protein dynamics, and conduct high-throughput antiviral screens and viral neutralisation assays (reviewed in [45]). However, the insertion of a reporter gene within a viral genome almost always attenuates viral fitness, which can lead to the rapid development of variants that no longer encode the reporter gene. Common approaches to mitigate the attenuation of orthoflaviviruses and hepaciviruses include the insertion of a reporter gene near an internal ribosome entry site (IRES) within the 5′ or 3′ UTRs of the viral genome, or the insertion of a reporter flanked by 2A self-cleaving peptides often immediately downstream of a small duplicated sequence within the 5′ end of the viral genome. These reporter viruses allow for the simultaneous expression of the reporter and viral genome and, most notably, have been employed in combination with high-throughput fluorescence imaging in functional genomics and antiviral drug screens to directly visualise flavivirus infection levels [46,47,48]. This review will instead focus on reporter viruses that harbour insertions within viral protein-coding regions as these enable sensitive monitoring and quantitative analysis of viral protein localisation, trafficking, and interactions within live infected cells. Genetically encoded reporters used for this purpose include fluorescent protein tags (e.g., eGFP and mCherry), self-labelling enzyme tags (e.g., SNAP-tag and HaloTag), and the tetracysteine (TC) tag (Table 3).

One approach to minimise the attenuation and genetic instability of reporter viruses is the identification of sites within viral genomes at which insertions are best tolerated. Several studies have employed random transposon-mediated mutagenesis to reveal sites that are permissive to insertions, enabling the generation of epitope- and reporter-tagged viruses or replicons for DENV, ZIKV, and HCV for use in various imaging and molecular applications [14,49,50,51,52]. Notably, Eyre et al. used a high-throughput transposon mutagenesis-coupled next-generation sequencing (NGS) approach to guide the insertion of the mScarlet red fluorescent protein within the NS1 gene of the full-length DENV2 genome (strain 16681), generating the functionally intact, replication-competent, and infectious DENV2-NS1-mScarlet reporter virus [14]. Live-cell imaging of DENV2-NS1-mScarlet-infected cells resulted in the detection of intensely fluorescent juxtanuclear NS1-mScarlet foci that remained static, which likely represents NS1 localised to virus-induced replication complexes. Weakly fluorescent NS1-mScarlet foci that exhibited rapid and bidirectional trafficking were also observed, which may represent NS1 proteins that are transported via the cytoskeleton. These observations highlight the applicability of DENV2-NS1-mScarlet to the elucidation of host cell factors and cellular pathways that enable NS1 to perform its functions and the mechanisms underlying the secretion of this protein. Li et al. generated a novel ZIKV reporter virus enabling the live-cell imaging of single virus particles [19]. Analysis of viral protein expression levels and virus particle assembly and release revealed that TC tag insertion was best tolerated at one of six candidate insertion sites, AA27/28 (TC27), within the capsid-encoding region of the ZIKV genome (strain Natal-RGN). The TC-tagged virus, NR-TC27, displayed mildly attenuated viral replication and infectivity, was genetically stable over multiple passages, and displayed unaltered capsid protein localisation. The fluorescent labelling of TC-tagged proteins involves the incubation of infected cells with membrane-permeant FIAsH (green fluorescent) or ReAsH (red fluorescent) biarsenical dyes which bind to the TC motif with high affinity. Real-time tracking of single ZIKV particles was achieved via the direct labelling of purified NR-TC27 particles with ReAsH or FIAsH to elucidate the mechanism by which ZIKV enters cells of the blood–brain barrier. It was shown that ZIKV particles attach to and traffic along the filopodia of human cerebral microvascular endothelial cells prior to entering the cells via endocytosis.

Reporter viruses have greatly improved our understanding of the HCV replication cycle. The HCV NS5A protein has been a major focus of live-cell imaging studies as it is a critical component of RCs and is essential for both viral replication and infectious virus particle assembly. Full-length viruses and subgenomic replicons harbouring insertions within NS5A have revealed new details regarding the formation, maturation, distribution, and motility of putative RCs [15,16,17,18,22,51]. In an early study by Wölk et al., live-cell wide-field and spinning disc confocal imaging of cells harbouring a fluorescent subgenomic replicon, combined with quantitative analysis of NS5A-GFP puncta fluorescence intensity, size, and velocity, revealed two distinct NS5A subclasses [15]. Intensely fluorescent, large NS5A-GFP puncta localised to the perinuclear region and remained static, whilst smaller cytosolic NS5A-GFP puncta either remained static or displayed rapid saltatory microtubule-dependent trafficking over long distances. Both subclasses co-localised with HCV NS3 puncta and ER tubules, supporting the hypothesis that large and static NS5A-GFP puncta represent RCs within membranous webs, whilst small and motile NS5A-GFP puncta may represent early RCs. Subsequent studies utilising NS5A reporter viruses have captured these two subclasses and demonstrated that NS5A co-localises with additional viral and host factors [16,22]. To study NS5A localisation and traffic in the context of the complete HCV life cycle, Eyre et al. generated replication-competent and infectious reporters by inserting a TC tag or GFP into NS5A within the full-length genotype 2a (J6CF/JFH1) HCV chimaera, Jc1 [16]. Live-cell wide-field fluorescence imaging showed that both ReAsH-labelled NS5A-TC and NS5A-GFP puncta, regardless of size or motility, strongly co-localised and co-trafficked with fluorescently tagged host factors VAP-A and Rab5A, which are involved in HCV RNA replication. NS5A motility was shown to be dependent upon the microtubule network and dynein cytoskeletal motor protein. The inhibition of microtubule dynamics abrogated the trafficking of FIAsH-labelled NS5A-TC puncta along α-tubulin-RFP-labelled microtubules, whilst the siRNA-mediated knockdown of dynein impaired the long-range trafficking of NS5A-GFP foci. Pulse-chase imaging of SNAP-tagged HCV NS5A reporter viruses with spectrally distinct fluorescent SNAP ligands has also been used to characterise temporally distinct NS5A subclasses [16,22]. Eyre et al. demonstrated that pre-existing and newly synthesised SNAP-tagged NS5A foci both consist of largely static and highly motile subclasses, although pre-existing foci were larger. Co-localisation between pre-existing and newly synthesised NS5A foci was shown to be infrequent. A subsequent study employing pulse-chase labelling of NS5A foci also demonstrated that putative RCs are continuously generated de novo at sites that are spatially distinct from pre-existing sites [22]. Pulse-chase imaging of SNAP-tagged NS5A foci relative to host cell membrane structures, cholesterol, and host factors involved in lipid metabolism revealed that PI4KA and OSBP are required for the formation and maintenance of NS5A foci [22]. Additionally, it was demonstrated that cholesterol preferentially accumulates in pre-existing NS5A foci and that this is a pre-requisite for their gradual recruitment to LDs.

Reporter viruses generated to study HCV structural protein dynamics have provided mechanistic insights into infectious virus particle assembly and release [18,20,21,23]. Infectious HCV reporters encoding a TC tag fusion to the core protein have been generated by two groups [20,21]. In both studies, TC tag insertion and biarsenical dye labelling minimally impacted replicative fitness and infectivity, and did not impair core protein function. However, the FIAsH/ReAsH fluorescent signals photobleached quickly, limiting the live-cell imaging to short durations. Counihan et al. inserted the TC tag near the *N*-terminus of the core protein in a full-length HCV cDNA clone (strain Jc1), producing the Jc1/core(TC) reporter virus [20]. To characterise the dynamics of core protein recruitment to cytosolic lipid droplets (cLDs), which is critical to virus particle assembly, cells stably expressing a cLD-associated protein fused to a fluorescent protein were infected with Jc1/core(TC). Live-cell spinning disc confocal imaging revealed that TC-tagged core proteins traffic from the ER to the surface of cLDs where they form static caps. Pulse-chase imaging with FIAsH and ReAsH allowed for the long-term dynamics of core trafficking to be imaged over short acquisitions, revealing that core proteins are slowly recruited from cLDs to motile TC–core puncta. The quantification of pulse-chase-labelled LD-associated TC–core and motile TC–core puncta in cells infected with a panel of Jc1/core(TC) mutants demonstrated that the interaction between HCV non-structural proteins 2 (NS2) and 3-4A (NS3-4A) is essential for the recruitment of core proteins from cLDs to motile TC–core puncta. Further live-cell imaging of Jc1/core(TC)-infected cell lines individually expressing fluorescent protein-tagged β-tubulin and actin revealed that the transport of motile TC–core puncta was microtubule-dependent. Coller et al. similarly generated a TC–core reporter virus by inserting the TC tag after the third amino acid of the core gene within the J6/JFH1 chimeric HCV genotype 2a infectious clone [21]. Live-cell spinning disc confocal imaging and quantitative analysis of TC–core protein velocity revealed that motile TC–core puncta frequently co-trafficked with fluorescently tagged microtubules, recycling endosome components, apolipoprotein E (ApoE), and VAMP1-associated secretory vesicles. Furthermore, live-cell imaging of cells infected with a NS2-deletion TC–core mutant incapable of infectious virus particle production revealed that viral particle assembly is a pre-requisite of TC–core puncta motility. Collectively the findings of Counihan et al. and Coller et al. support the hypothesis that motile core puncta represent virus particles undergoing canonical Golgi-mediated secretion. Subsequent imaging studies of HCV particle assembly and release employed reporter viruses encoding fluorescent protein insertions within the HCV envelope glycoproteins 1 and 2 (E1 and E2) [18,23]. Bayer et al. generated an HCV Jc1 reporter virus harbouring an mCherry insertion within E1 [18]. Use of this reporter in proximity ligation assays demonstrated that the interaction of E1-mCherry with E2 and core proteins increases with time, suggesting that E1-mCherry puncta may represent virus particles undergoing assembly and secretion. Immunofluorescence assays and fluorescence recovery after photobleaching (FRAP) experiments indicated that E1-mCherry punctae co-localise with ER and endosomal markers as opposed to Golgi markers, leading the authors to hypothesise that HCV particles are transported within endosomal compartments and are released via the non-canonical secretory route. Although no major defects in structural protein localisation and expression, viral RNA replication, or the composition and secretion of virus particles were identified, both reporter viruses displayed strongly attenuated infectivity and thus may not provide an accurate representation of viral assembly events.

**Figure 3:**
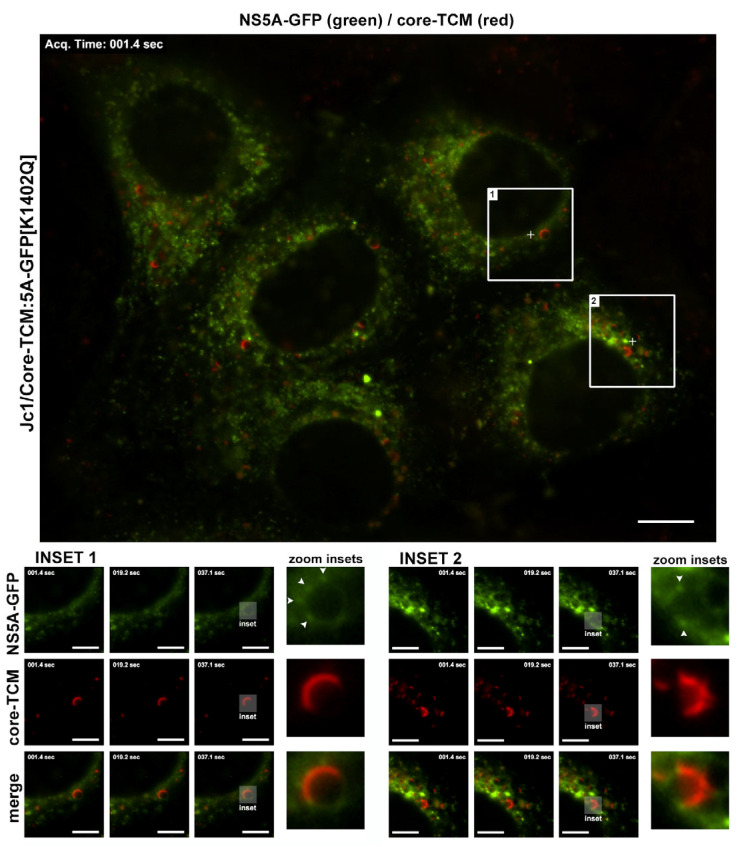
Localisation of HCV core and NS5A proteins in live infected Huh-7.5 cells. Cells were electroporated with in vitro transcribed RNA of the dual-tagged full-length HCV reporter virus, Jc1/Core–TCM:5A-GFP [K1402Q], encoding a TC tag within core and GFP insertion within NS5A. ReAsH labelling of core–TCM was performed at 48 h post-transfection and live-cell imaging was performed at 72 h post-electroporation. Insets 1 and 2 highlight the stable association of a small proportion of NS5A-GFP foci with core–TCM at the LD surface. Figure reproduced from Eyre et al. (2014), with permission from the Journal of Virology [16].

Dual-tagged HCV reporter systems have provided new insights into the spatiotemporal organisation of viral replication and assembly [16,18,23]. To resolve whether NS5A interacts with core at the surface of lipid droplets (LDs), which is thought to facilitate the transfer of viral RNA from RCs to core-coated LDs for encapsidation, Eyre et al. generated a dual-tagged full-length HCV reporter virus encoding a TC tag within core and GFP insertion within NS5A [16]. Live-cell imaging revealed that a small proportion of NS5A-GFP foci co-localised with ReAsH-labelled core–TCM foci at LD surfaces and remained relatively static, suggesting that interactions between NS5A and core are infrequent (Figure 3). A dual-tagged HCV (strain Jc1) reporter virus harbouring a GFP insertion within NS5A and an mCherry insertion within E1 was also generated by Bayer et al. [18]. Live-cell spinning disc confocal imaging conducted every 24 h over a 96 h period revealed that NS5A-GFP punctae initially accumulated rapidly and then began to decline at 72 h post-electroporation, whereas E1-mCherry puncta steadily accumulated over time. Lee et al. generated the Jc1/^egfp-CS^E2 HCV reporter virus by direct fusion of eGFP and a human rhinovirus 3C protease cleavage site to the *N*-terminus of envelope glycoprotein E2 [23]. Upon release of the Jc1/^egfp-CS^E2 particles into a cell culture supernatant containing PreScission Protease (PSP), eGFP is removed from E2, significantly improving viral infectivity. A trans-complementation system was established in which cells stably expressing C-NS2/^egfp-CS^E2 were transfected with an HCV subgenomic replicon encoding mCherry-tagged NS5A (sgrJFH/5A^mCherry^) to produce infectious virus. Utilising this system, the authors demonstrated that the E2-NS5A double-positive puncta proximal to the surface of LDs likely represent HCV assembly sites. Live-cell spinning disc confocal imaging of these cells every 15 min over a 16 h period captured the spatiotemporal dynamics of the viral replication-dependent relocalisation of E2 from the ER to LDs to which NS5A concurrently localised. Both E2 and NS5A displayed restricted motility following co-localisation at the LD surface. Correlative light and electron microscopy (CLEM) was used to determine the ultrastructure of E2-NS5A double-positive puncta within sgrJFH/5A^mCherry^-electroportated C-NS2/^egfp-CS^E2 cells and Jc1/^egfp-CS^E2-infected cells, indicating that RCs are exclusively formed in close proximity to the putative assembly sites to enable efficient packaging of newly synthesised genomes into virus particles.

## 5. Visualisation of Virus Binding and Entry Using Fluorescently Labelled Virus Particles

Real-time imaging of individual virus particles during the early stages of an infection has proven to be a valuable approach to studying the dynamic virus–host interactions that constitute viral entry. This has predominantly been achieved through the fluorescent labelling of purified virus particles with bright and highly photostable lipophilic dyes. Lipophilic dyes emit a fluorescent signal once inserted within the viral lipid bilayer membrane and are retained within the endosomal compartment following membrane fusion [53]. DiD (1,1′-dioctadecyl-3,3,3′,3′-tetramethylindodicarbocyanine), a deep-red lipophilic dye, has been extensively used for the purpose of single virus particle tracking to contrast blue cell autofluorescence [53]. When inserted within the viral lipid bilayer membranes at high densities, DiD self-quenches, resulting in the detection of weaker virus particle-associated fluorescent signals [54]. This property has been exploited to discern viral membrane fusion events as lipophilic dyes de-quench once inserted within the larger endosomal membrane following its fusion with the viral membrane [24,54]. As a brighter alternative to lipophilic dyes, amine-reactive succinimidyl ester fluorescent dyes can be used to label purified virus particles (Table 3). The simultaneous visualisation of fluorescently labelled virus particles and cellular markers by multi-colour live-cell imaging has offered pivotal insights into the mechanisms and kinetics underlying virus particle attachment, internalisation, membrane fusion, and endosomal trafficking. These insights are largely informed by the computational construction and quantitative analysis of the trajectories of imaged single virus particles (reviewed in [55]). Information that can be obtained from trajectory analysis includes instantaneous speed, instantaneous and average velocity, net and total distance travelled, and mean square displacement (MSD) [55]. Changes in DiD-associated fluorescence intensity with respect to time can also be determined to identify peaks in intensity that represent fusion events [55]. Importantly, fluorescent dyes were shown to have little to no effect on viral infectivity in the studies discussed in this section.

Protocols for labelling DENV particles with lipophilic DiD or amine-reactive succinimidyl ester fluorescent dyes are well established [24,54,56,57,58]. In this context, Van der Schaar et al. used live-cell imaging of DiD-labelled DENV particles in cells expressing fluorescent markers for clathrin, early endosomes, and late endosomes to study DENV internalisation [24]. DENV particles were observed to bind to the surface of host cells and slowly move towards clathrin-coated pits in a diffuse manner, indicating that the particles bind multiple attachment factors before engaging entry receptors within clathrin-coated pits. Clathrin signals around DENV particles intensified, indicative of vesicle formation and budding, and transported the particles unidirectionally to the perinuclear region. Thereafter, clathrin was observed to dissipate as the vesicles uncoated and DiD fluorescence dequenched following the fusion of viral and endosomal membranes. Entry and fusion events were not detected following the treatment of cells with clathrin inhibitors, confirming that DENV particles are internalised exclusively via clathrin-mediated endocytosis. Moreover, following entry, DENV particles were delivered to either Rab5-positive early endosomes or Rab5/Rab7-postive intermediate endosomes. These endosome populations matured into Rab7-positive late endosomes which served as the primary cellular location of viral membrane fusion. Using a complementary protein-labelling approach, Zhang et al. established a protocol to covalently tag the amine groups of the DENV envelope glycoprotein with Alexa Fluor 594 succinimidyl ester red fluorescent dye [58]. Owing to the high unquenched brightness and photostability of the Alexa Fluor 594 succinimidyl ester dye, this approach can confer enhanced visibility of early entry events and virus–host interactions in comparison to labelling with lipophilic dyes. Live-cell imaging of Alexa Fluor 594-labelled DENV2 particles by Total Internal Reflection Fluorescence (TIRF) microscopy demonstrated that the ubiquitinated T-cell immunoglobulin mucin-1 (TIM-1) phosphatidylserine receptor mediates DENV endocytosis [28]. Trajectory analysis revealed that Alexa Fluor 594-labelled DENV2 particles and GFP-tagged TIM-1 receptors are co-internalised via clathrin-mediated endocytosis. DENV entry was abrogated within TIM-1 knockout and clathrin heavy-chain knockdown cells and severely reduced within cells stably expressing TIM-1 mutants lacking the lysine residues within its cytoplasmic tail that serve as ubiquitination sites.

Another lipophilic dye, octadecyl rhodamine B chloride (R18), was used to label YFV particles and JEV non-infectious virus-like particles (JE-VLPs) at self-quenching concentrations to study the dynamics of viral membrane fusion and nucleocapsid release [25]. Live-cell imaging of R18-labelled YFV particles or JE-VLPs in cells treated with inhibitors suggested that both flaviviruses fuse within intermediate endosomes which subsequently mature to late endosomes before the release of the nucleocapsid. Furthermore, fluorescent labelled ZIKV particles have been used to study the mechanisms by which ZIKV penetrates the placental and blood–brain barrier to infect the foetal central nervous system (CNS) [19,29]. In a study by Chiu et al., ZIKV particles were labelled by tagging the envelope glycoprotein with Atto647N-NHS ester dye [29]. Upon live-cell imaging, Atto647N-labelled ZIKV particles were observed to transit from the apical side to the basolateral side of polarised human placental trophoblast-derived cell (JEG-3) and human cerebral microvascular endothelial cell (hCMEC/D3) monolayers, which serve as models of the placental and blood–brain barrier, respectively. These observations contributed towards the finding that ZIKV penetrates the placental and blood–brain barrier via transcytosis. The treatment of JEG-3 and hCMEC/D3 cells with inhibitors of endocytosis blocked ZIKV transcytosis, complementing the results of a study by Li et al. in which ZIKV particles that were sequentially labelled with FIAsH and DiD entered hCMEC/D3 cells via endocytosis [19].

The current model of HCV entry proposes that lipoprotein-associated HCV particles attach to glycosaminoglycans (GAGs), the low-density lipoprotein receptor (LDL-R), and the scavenger receptor class B type I (SR-BI) at the surface of hepatocytes. This results in the exposure of E2 glycoprotein domains, which allows for binding to CD81 tetraspanin molecules. CD81-bound HCV particles then migrate to tight junctions where CD81 associates with claudin-1 (CLDN1) and occludin (OCDN), triggering the clathrin-mediated endocytosis of HCV particles. The tight junction protein occludin (OCDN) is also essential for HCV entry, though the precise role of this protein has not yet been identified [33]. Studies utilising lipophilic dyes have visualised the sequence in which HCV particles engage multiple host cell entry factors and have shown that the transport of virus particles during entry and internalisation is actin-dependent [26,27]. Coller et al. performed live-cell spinning disc confocal imaging of DiD-labelled HCV particles in cells transiently expressing GFP-actin [26]. This revealed that DiD-labelled HCV particles migrate towards the cell surface in association with filopodia at a speed consistent with retrograde actin flow. At the cell surface, actin filaments surrounded the particles, which may represent actin clustering during clathrin-mediated endocytosis. Internalised DiD-labelled HCV particles migrated towards and trafficked along actin stress fibres. Additionally, DiD-labelled HCV particles co-localised with immuno-labelled clathrin light chain and the E3 ubiquitin ligase c-Cb1, which mediates receptor internalisation, and are co-trafficked with Rab5-positive early endosomes in GFP-Rab5 expressing cells. An orange lipophilic dye, Dil (1,1′-dioctadecyl-3,3,3′,3′-tetramethylindocarbocyanine perchlorate), was also used in this study to track single HCV particles in CD81-GFP expressing cells. The Dil-labelled HCV particles migrated towards the cell surface, co-localised with CD81-GFP, and were then internalised along with CD81-GFP at the cell surface. The authors proposed that internalisation occurred outside tight junctions due to the unpolarised nature of two-dimensional Huh-7.5 cell monolayers, as evidenced by CLDN1 and the tight junction marker ZO-1 being distributed across the plasma membrane. It was also recognised that HCV may disrupt tight junction barrier function, causing the mislocalisation of CLDN1 and OCLN. In a subsequent study, polarised three-dimensional hepatoma organoids were infected with DiD-labelled HCV particles and individually immunolabeled for host entry factors [27]. Importantly, ZO-1, CLDN1, and OCLN exclusively localised within tight junctions, which remained unperturbed during HCV entry. This study demonstrated that DiD-labelled HCV particles initially co-localise with SR-BI, CD81, and the epidermal growth factor receptor (EGFR) at the basolateral membrane. The DiD-labelled HCV particles were then shown to accumulate within tight junctions where they co-localise with CLDN1 and OCLN, before being internalised via EGRF-dependent clathrin-mediated endocytosis. Furthermore, the migration of DiD-labelled HCV particles from the basolateral membrane to tight junctions was shown to be dependent on actin, corroborating the findings of Coller et al. [26].

## 6. Imaging Viral RNA Localisation and Traffic Using Genetically Tagged and Wildtype Viral Genomes

Real-time imaging of viral RNA localisation and trafficking can provide insight into how viruses spatially and temporally coordinate viral genome replication, translation, and packaging into virus particles and can be used to identify and characterise interactions occurring between the viral genome and viral or host proteins. Several approaches have been developed to detect RNA within live mammalian cells with single-molecule resolution (reviewed in [59]). The approach that is most frequently used to image RNA in living cells is the MS2-MCP system, which exploits the ability of the bacteriophage MS2 coat protein (MCP) to bind to the MS2 21-nt RNA stem-loop encoded in the phage genome with high affinity (Table 3). This system involves the co-expression of MS2 fused to a fluorescent protein or self-labelling enzyme tag (SNAP-tag or HaloTag), and an exogenous RNA molecule of interest which has been genetically modified to encode multiple copies of the MS2 stem-loop sequence. Similarly, the bacteriophage PP7 and λN RNA-binding proteins have been used to develop analogous RNA imaging systems [59]. The main drawbacks of these systems are the high level of background signal produced by unbound reporter proteins and the related need to incorporate multiple copies of the relevant RNA stem-loops in an RNA molecule of interest to achieve a strong signal-to-noise ratio (SNR). In the case of the MS2-MCP system, the insertion of 24 copies of the MS2 stem-loop is required to achieve an SNR that permits single-molecule resolution. The fusion of MCP to split fluorescent protein fragments or split HaloTag pairs and the addition of nuclear localisation signals to MCP have been shown to decrease background signals and improve the SNR [60,61]. Utilisation of the MS2-MCP system in combination with the SunTag fluorescent signal amplification system has also been shown to increase the signal-to-noise ratio for RNA imaging [62,63,64].

Fluorogenic RNA aptamers are another class of genetic tag that can be used to visualise RNA in live cells. Cells expressing the aptamer-tagged RNA of interest are treated with a cell-permeant conditionally fluorescent small molecule to which the aptamer binds with high affinity, producing a stabilisation effect which activates the fluorescence of the small-molecule [59]. Although such systems produce low background fluorescence, their utility is generally limited by the poor brightness and photostability of the fluorescent small molecules. An alternative approach that enables the imaging of endogenous non-genetically modified RNA in live cells utilises aptamers capable of hybridising to an RNA molecule of interest in combination with conventional fluorophores conjugated to a quencher to increase brightness and photostability whilst producing minimal background fluorescence (Table 3). This system can be constructed such that the hybridisation of the aptamer to the target RNA is required to enable it to bind a cell-permeant–fluorophore-quencher conjugate. Aptamers that are conjugated to a fluorophore-quencher pair, termed molecular beacons, have also been developed and applied towards imaging of *Flaviviridae* virus replication [59]. In both cases, the binding of the aptamer to the target RNA separates the fluorophore from the quencher, restoring its fluorescence. These large MS2 tags and fluorogenic RNA aptamers are not always tolerated and have often been shown to perturb replication. This is likely due to the disruption of secondary RNA structures promoting the circularisation of the orthoflavivirus RNA genomes, which is critical to the initiation of viral genome replication [65]. Over recent years, several groups have applied the CRISPR-Cas13 system to the visualisation of endogenous RNA in live mammalian cells [66,67,68,69]. This system generally involves the co-transfection of cells with plasmids that encode multiple CRISPR RNAs (crRNAs) each complementary to a specific region of an RNA molecule of interest, and plasmids encoding endonuclease-deficient RNA-targeting Cas13 RNases (dCas13) fused to a fluorescent protein such as eGFP. When expressed, crRNAs bind to a dCas13-eGFP fusion protein and hybridise to the targeted RNA molecule, enabling the detection of RNA-associated fluorescence. While challenging, the application of this approach to the live-cell imaging of *Flaviviridae* family virus RNA is promising.

Miorin et al. were the first to visualise positive single-stranded viral RNA in live cells [70]. This group generated a TBEV subgenomic replicon encoding an array of 24 MS2 binding sites within the 3′ untranslated region, enabling the visualisation of TBEV RNA in cells expressing MCP fused to an enhanced yellow fluorescent protein (EYFP) and NLS. FRAP and fluorescence loss in photobleaching (FLIP) analysis was performed to track the movement of TBEV RNA within and between virus-induced replication organelles, identified as EYFP-MCP-enriched sites, within the perinuclear region and the cytoplasmic extravesicular space [30]. This revealed that, in addition to static ER membrane-associated viral RNA, virus-induced replication organelles contain a motile viral RNA fraction, the egress of which into the extravesicular space is restricted. Immunogold electron microscopy of EYFP-MCP and TBEV subgenomic replicon-transfected cells labelled with anti-GFP antibodies showed that newly synthesised viral RNA genomes predominately localised to the extravesicular space surrounding virus-induced membrane alterations. Together with electron microscopy-based imaging of the ultrastructural organisation of viral replication organelles, these findings led the authors to propose that TBEV genomes are synthesised within the replication organelles and then released into an extravesicular space confined by a network of interconnected virus-induced ER membrane rearrangements to be translated or packaged within virus particles. The MS2-MCP system has also been applied to the study of HCV RNA genome localisation and traffic relative to cLDs and the HCV NS5A protein [31]. To simultaneously visualise the RNA genome and NS5A protein localisation dynamics in live cells, an array of 24 MS2 binding sites was inserted within the 3′ untranslated region of a full-length HCV (strain Jc1) reporter virus harbouring a TC tag within NS5A. Whilst the insertion of MS2 repeats did not impact replicative fitness, infectious virus particle production and/or cell-to-cell spread was significantly attenuated. Cells stably expressing MCP-mCherry were electroporated with Jc1/5A-TCM+3′UTR:24xMS2 RNA. Live-cell imaging of these cells resulted in the detection of two distinct subclasses of HCV RNA puncta which correspond with the two subclasses of NS5A puncta observed in previous live-cell imaging studies [15,16,22]. This included small and highly motile puncta that displayed sporadic and long-range bidirectional trafficking throughout the cytoplasm, or large and static puncta that predominately localised within the perinuclear region of the cytoplasm. Both subclasses of RNA puncta co-localised with FIAsH-labelled NS5A-TC puncta, suggesting that they may represent RCs or RNAs that are in transit to cLDs to undergo encapsidation. To visualise HCV RNA localisation and traffic to cLDs, MCP-mCherry expressing cells were electroporated with Jc1/5A-TCM+3′UTR:24xMS2 RNA and treated with the BODIPY 493/503 lipophilic dye. HCV RNA puncta trafficked towards and associated with the surface of cLDs in a formation that closely resembles the HCV core- and NS5A-positive cap-like structures seen on the surface of cLDs in previous live-cell imaging studies [16,21].

## 7. Considerations in Live-Cell Imaging Approaches

**Figure 4:**
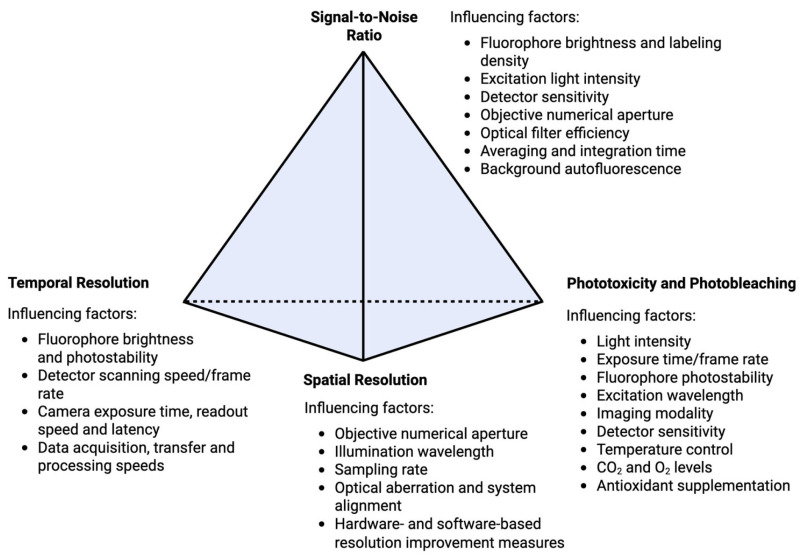
Pyramid of frustration in fluorescence microscopy. Each vertex represents a core imaging constraint of signal-to-noise ratio, phototoxicity and photobleaching, spatial resolution, and temporal resolution. The key influencing factors are listed for each constraint. Improvements in one area will often compromise the others.

Live-cell imaging requires cells to be exposed to high-intensity light for prolonged periods to capture the spatiotemporal dynamics of cellular components at a high resolution. Prolonged exposure to high intensity light can cause phototoxicity, which includes the induction of oxidative stress, direct damage to cellular structures and DNA, and apoptosis [71]. It can also cause photobleaching, where fluorophores lose the ability to fluoresce. Several strategies can be implemented to minimise phototoxicity and photobleaching, in turn prolonging the duration over which high-quality, high-resolution images of viable live cells can be obtained (reviewed in [72]; Figure 4). This has largely been achieved through the advancement of conventional and super-resolution microscope optical systems. Light-emitting diodes (LEDs) and lasers that emit light at specific wavelengths to precisely match fluorophore absorption peaks have improved excitation efficiency and SNR whilst also reducing the intensity of light required. Light intensity requirements are further reduced through the optimisation of the objective lens numerical aperture and coating such that light is focused on the target area with greater precision and then gathered with minimal losses. Furthermore, highly sensitive high-speed detectors have been developed to improve SNR, increase temporal resolution, and reduce the duration of light exposure that is required. The majority of the live-cell imaging studies of aspects of orthoflaviviral and hepaciviral replication cycles described above have been performed using ‘traditional’ spinning disc confocal microscopy (SDCM), laser scanning confocal microscopy (LSCM), and wide-field fluorescence microscopy approaches. However, the future application of recently developed live-cell imaging techniques that enable improvements in spatial resolution, temporal resolution, and/or reduced exposure of cells to damaging light, such as advanced structured illumination microscopy (SIM) and lattice light sheet microscopy systems and associated image processing/reconstruction algorithms (reviewed in [73]), may reveal new details of viral replication cycles and virus–host interactions that have not been appreciated to date (Table 4).

Advances in fluorophores have also enhanced live-cell imaging with higher resolution and reduced phototoxicity. The continual development of new variants of genetically engineered fluorescent proteins with improvements in properties such as brightness, photostability, maturation rates, behaviour as fusion proteins, resistance to chemical fixation, and more desirable excitation/emission spectra can greatly enhance live-cell and fixed-cell imaging studies. For example, the recent development of extremely photostable and bright fluorescent proteins, such as mStayGold and mScarlet, have facilitated increases in the spatiotemporal resolution of three-dimensional live-cell imaging experiments that are not typically viable using traditional green and red fluorescent proteins such as EGFP and mCherry [75,76,77,78,79]. In particular, photostable fluorophores resist degradation and photobleaching, reducing the need for high-intensity illumination. Similarly, improvements in organic fluorophores that can be used in the live imaging of proteins that have been fused to self-labelling enzymes (HaloTag and SNAPtag), such as the JaneliaFluor series of fluorescent dyes, have facilitated the application of super-resolution imaging techniques that typically require high laser powers and/or extended exposure to excitation light, such as STED, PALM and dSTORM, for live-cell imaging [80,81,82,83]. While not the focus of the present review, the interested reader is directed to a current and comprehensive review of super-resolution microscopy techniques and their respective strengths and weaknesses authored by Prakash et al. [74]. Improved fluorophores enable live-cell imaging studies with reduced levels of oxidative stress, emphasising the importance of selecting suitable fluorescent proteins for imaging. The biological model selected for live-cell imaging can also influence phototoxicity. In comparison to primary cell lines, immortalised cell lines have been shown to exhibit a greater tolerance to light exposure [72]. Oxidative stress may be further mitigated through the precise control of the temperature, CO_2_, and oxygen levels within the imaging chamber, as well as the supplementation of cell culture medium with antioxidants [72].

## 8. Future Directions

The development of live-cell fluorescence imaging approaches and biological tools has greatly enhanced our understanding of orthoflaviviral and hepaciviral life cycles, allowing researchers to measure infection kinetics and characterise the spatiotemporal regulation of distinct stages of the viral life cycles, as well as the viral and host cell determinants that are critical to these stages. These approaches and biological tools can serve as useful models to guide the study of emerging viruses within the *Flaviviridae* family, unrelated viruses, viral evolution in response to selective pressures, and the effects of antiviral drug treatments. The development of live-cell multi-colour imaging approaches that enable the simultaneous visualisation of multiple virus and host cell components, and thus multiple stages of the virus life cycle, is warranted. This is challenged by the difficulty of generating replication-competent reporter viruses that harbour multiple reporter genes, and the need for spectrally distinct, small, bright, and highly photostable fluorophores. Future studies of the spatiotemporal dynamics of viral RNA molecules at distinct stages of the viral life cycle would greatly benefit from the identification of an approach that permits the long-term and multiplexed live-cell imaging of unperturbed viral RNA relative to other viral and host cell components. Furthermore, the future utilisation of advanced microscopy approaches, including super-resolution microscopy approaches that are compatible with live-cell imaging (reviewed in [84]), would enable the study of viral protein localisation and trafficking relative to that of host proteins and sub-cellular structures with nanoscale spatiotemporal resolution. Together, such studies will unveil new and precise details about the replication cycles of these viruses and therefore contribute to the future development of antiviral strategies.

## Figures and Tables

**Table 1:** Summary of the live-cell imaging approaches developed to study the *Flaviviridae* virus family members.

Approach	Labelling Strategy	Stage of Viral Life Cycle Investigated	Virus, Tag Location, and References
Monitoring Viral Replication and Spread Using Viral Protease-Dependent Reporters	Viral protease-dependent translocation of GFP from the ER or mitochondria to the nucleus of infected cells.	Monitoring viral replication and spread	DENV [7,8], ZIKV [9], HCV [10] and Flavivirus [11]
	Viral protease-dependent translocation of a transactivator or Cre recombinase to the nucleus of infected cells to trigger GFP or mCherry expression.	Monitoring viral replication and spread	DENV [12] and HCV [13]
Genetic Tagging of Viral Genomes for Analysis of Viral Protein Traffic and Virus–Host Interactions	Genetically encoded fluorescent proteins (GFP, mScarlet, and mCherry)	Replication	DENV NS1 [14]
		Replication and RC biogenesis	HCV NS5A [15,16,17]
		Virus particle assembly and release	HCV E1 [18]
	Genetically encoded tetracysteine (TC) tag	Entry	ZIKV Capsid [19]
		Replication	HCV NS5A [16]
		Virus particle assembly and release	HCV Core [20,21]
	Self-labelling enzyme tags (SNAP-tag)	Replication and RC biogenesis	HCV NS5A [16,22]
	Dual genetic tags (two fluorescent proteins or one fluorescent protein and one TC tag)	Spatiotemporal organisation of replication and virus particle assembly	HCV NS5A and Core [16], NS5A and E1 [18], or NS5A and E2 [23]
Visualisation of Virus Binding and Entry Using Fluorescently Labelled Virus Particles	Lipophilic dyes (DiD, Dil, and R18)	Attachment and entry	DENV [24], YFV [25], JEV-VLP [25], and HCV [26,27] lipid bilayer membrane
	Amine-reactive succinimidyl ester fluorescent dyes (Alexa Fluor 594 NHS ester and Atto647N-NHS ester)	Attachment and entry	DENV [28] and ZIKV [29] envelope
Imaging Viral RNA Localisation and Traffic Using Genetically Tagged and Wildtype Viral Genomes	Genetically encoded MS2 binding sites	Replication	3′ UTR of the TBEV [30] or HCV [31] genome

**Table 2:** Strengths and limitations of the live-cell imaging approaches developed to study the *Flaviviridae* virus family members.

Approach	Strengths	Limitations
Monitoring Viral Replication and Spread Using Viral Protease-Dependent Reporters	Real-time monitoring of viral infections with single-cell resolution. Applicable to high-throughput screening of antivirals. High specificity. Easily modified to encode a different cleavage site.	Reporters rely on the overexpression of fluorescently tagged reporter constructs that are protease-sensitive and may alter viral replication cycles and/or virus–host interactions.
Genetic Tagging of Viral Genomes for Analysis of Viral Protein Traffic and Virus–Host Interactions	Visualisation of viral protein localisation, trafficking, and interactions with high spatiotemporal resolution. Applicable to high-throughput screening of antivirals. Many reporter genes are available.	Insertion of a reporter gene within a viral genome may attenuate viral fitness and can perturb viral protein structure and function.
Visualisation of Virus Binding and Entry Using Fluorescently Labelled Virus Particles	Visualisation of viral particle localisation, trafficking, and interactions during attachment, entry, and internalisation with high spatiotemporal resolution. Does not require the genetic modification of viral genomes.	Labelling of virus particles may perturb viral infectivity. Limited to the study of very early events in the viral replication cycle.
Imaging Viral RNA Localisation and Traffic Using Genetically Tagged and Wildtype Viral Genomes	Visualisation of viral RNA genome localisation, trafficking, and interactions, potentially with single-molecule and high spatiotemporal resolution.	Insertion of foreign sequences within a viral genome may attenuate viral replicative fitness. Limited sensitivity due to background signals.

**Table 3:** Strengths and limitations of the fluorescent labelling strategies used to study the *Flaviviridae* virus family members.

Label	Labelling Strategy	Strengths	Limitations
Fluorescent proteins (e.g., GFP, mScarlet, mCherry)	Genetically encoded.	Highly specific labelling.	Large size, RNA perturbation, variable photostability and brightness, spectral overlap, and maturation time.
Self-labelling enzymes (SNAP-tag)	Genetically encoded. Catalyse the covalent attachment of membrane-permeant fluorescent dyes.	Highly specific labelling, better photostability, high brightness, flexible, and immediate fluorescence after fluorescent dye labelling.	Large size and the timing of dye labelling requires consideration.
Tetracysteine (TC) tag	Genetically encoded. Bound by membrane-permeant biarsenical dyes (FIAsH and ReAsH).	Small size, flexible, self-labelling, and immediate fluorescence after dye binding.	Moderate brightness and photostability, few dyes available, and requires the application of harsh reducing agents to achieve specific labelling and improve SNR.
Lipophilic dyes (DiD, Dil, R18)	Hydrophobic insertion into lipid membrane.	Applicable for the study of virus attachment, entry, and lipid bilayer fusion events.	Variable photostability and brightness and the specific labelling of virus particles requires multiple laborious steps.
Amine-reactive succinimidyl ester fluorescent dyes (Alexa Fluor 594 NHS ester, Atto647N-NHS ester)	NHS ester covalently binds amine groups of proteins.	Applicable to the study of virus attachment and entry, high labelling stability, and high brightness and photostability.	Potential for off-target labelling and impacts on virus–host protein interactions and specific labelling requires multiple laborious steps.
MS2 binding sites	Genetically encoded. Bound by MS2 fusions to fluorescent proteins or self-labelling enzymes.	Highly specific labelling.	Large size, high background fluorescence, and variable photostability and brightness.
RNA aptamers	Hybridised to target RNA. Bound by membrane-permeant fluorophore–quencher pair.	Low background fluorescence.	Large size and variable photostability and brightness.

**Table 4:** Comparison of the spatial resolution, advantages, and limitations of the imaging modalities mentioned in this review. For a detailed and comprehensive review of these and other imaging modalities, refer to [74].

Imaging Modality	Best Spatial Resolution (Lateral: XY and Axial: Z)	Advantage (s)	Limitation (s)
Wide-field	XY: ~250 nm Z: ~500 nm	Inexpensive, high temporal resolution, and low photobleaching/phototoxicity.	Low spatial resolution.
Laser Scanning Confocal	XY: ~200 nm Z: ~500 nm	High spatial resolution.	Low temporal resolution and photobleaching/ phototoxicity.
Spinning Disc Confocal	XY: ~200 nm Z: ~500 nm	High spatial and temporal resolution.	Moderate photobleaching/ phototoxicity.
Lattice Lightsheet	XY: ~250 nm Z: ~300 nm	High temporal resolution, very low photobleaching/phototoxicity, long-term volumetric live-cell imaging, and near-isotopic resolution.	Moderate spatial resolution.
SIM	XY: ~100 nm Z: ~250 nm	Super resolution and low photobleaching/phototoxicity.	Moderately intensive data processing.
STED	XY: ~50 nm Z: ~100 nm	Super resolution.	Low temporal resolution and photobleaching/ phototoxicity.
dSTORM / PALM	XY: ~20 nm Z: ~60 nm	Super resolution with single molecule sensitivity.	Very low temporal resolution, photobleaching/phototoxicity, complex sample preparation (dSTORM), and intensive data processing.
